# Healthcare access and quality of birth care: narratives of women living with obstetric fistula in rural Tanzania

**DOI:** 10.1186/s12978-016-0189-x

**Published:** 2016-07-22

**Authors:** Lilian T. Mselle, Thecla W. Kohi

**Affiliations:** Department of Clinical Nursing, Muhimbili University of Health and Allied Sciences, PO Box 65004, Dar es Salaam, Tanzania; Department of Nursing Management, Muhimbili University of Health and Allied Sciences, PO Box 65004, Dar es Salaam, Tanzania

**Keywords:** Obstetric fistula, Emergency obstetric care, Women’s birth care, Narratives, Tanzania

## Abstract

**Background:**

Increasing births with skilled attendants and increasing health facilities with Emergency Obstetric Care (EmOC) can reduce maternal mortality and are considered critical interventions for ensuring safe motherhood. Despite Tanzania’s policy to support women to give birth with the assistance of skilled personnel, some women do not access this care. This article uses women’s stories to illustrate the challenges that caused them to fail to access adequate obstetric care in a timely manner, hence causing the development of fistulas.

**Methods:**

This paper presents the narratives of 16 women who were conveniently selected based on their experiences of not being able to access adequate obstetric care in timely manner. The analysis was guided by recommendations for the identification and interpretation of narratives, and identified important components of women’s experiences, paying attention to commonalities, differences and areas of emphasis. Semi-structured interviews were carried out at CCBRT hospital in Dar es Salaam.

**Results:**

Four (4) general story lines were identified from women description of their inability to access quality obstetric care in a timely manner. These were; failing to decide on a health care facility for delivery, lacking money to get to a health care facility, lacking transportation to a health care facility and lacking quality birth care at the health care facility.

**Conclusion:**

Women were unable to reach to the health care facilities providing comprehensive emergency obstetric care (CEmOC) in time because of their lack of decision-making power, money and transportation, and those who did reach the facilities received low quality birth care. Empowering women socially and financially, upgrading primary health care facilities to provide CEmOC and increased numbers of skilled personnel would promote health care facility deliveries.

## Background

Increasing births with skilled attendants and increasing the number of health facilities with Emergency Obstetric Care (EmOC) can reduce maternal mortality and are critical interventions for ensuring safe motherhood. These resources help ensure the timely provision of emergency obstetric and new-born care when life-threatening complications arise [1]. Approximately 60 % of maternal deaths occur during labour, delivery and the immediate postpartum period, with 50 % of these deaths occurring within the first 24 h of delivery [2]. Tanzania is a sub-Saharan African country with unacceptably high numbers of maternal morbidities, including obstetric fistula, and deaths, with an estimated maternal mortality ratio of more than 300 per 100,000 live births [3]. Maternal deaths are commonly caused by postpartum haemorrhage, obstructed labour, eclampsia, sepsis and abortion complications [4, 5], and previous studies have reported that approximately 15 % of all pregnant women will develop pregnancy and childbirth related complications that require prompt access to EmOC [6, 7].

Attendance to antenatal care, delivery in a medical setting and having skilled health personnel at delivery are key health care interventions that can largely prevent women from dying of pregnancy related causes and improve maternal and neonatal outcomes [8, 9]. While approximately 98 % of pregnant women attend antenatal clinics (ANC) for check-ups at least once, only 50 % give birth in health facilities, and among these, only 51 % are attended by skilled health personnel [10]. Tanzania’s government implemented various interventions to reduce maternal mortality, including reproductive and child survival, increased skilled delivery, maternal death audit, coordination and integration of different programs such as maternal and child health services, family planning, malaria interventions, expanded program on immunization and adolescent health and nutrition programmes. These initiatives are, however, challenged by an inadequate number of women who seek maternal health services because many women still give birth in homes. Women do not seek adequate obstetric care because of distance to the health facilities that provide EmOC, their low socio-economic status and cultural factors, institutional cost, limited material and supplies in the facility, attitudes and competence of health care providers, and lack of staff managerial support [6, 11–13].

Poor access to EmOC is associated with negative maternal and neonatal outcomes. One of the outcomes of poor access to EmOC during childbirth is disabling obstetric complications, the most disabling of which is obstetric fistula. Each year, obstetric fistula affects more than 100,000 women worldwide [14] and more than 2500 women and girls in Tanzania [15]. It occurs when labour is allowed to progress for periods lasting from several days to a week, where the foetal head exerts pressure on surrounding soft tissues, which results in necrosis and the concomitant creation of an opening through which urine and/or faeces leak [16].

At the personal level, women living with obstetric fistula experience multiple losses [17], including physical problems, social exclusion and economic decline [15, 18]. The disability that women acquire from the resulting smell, sores, infection, and inability to control the leaking of urine leave them unable to assume their ascribed social and marital roles [17]. Families and the community often exclude women living with fistulas [15, 18–20]. They experience a severe reduction in their independent income, which results in increased dependence on others [20, 21].

Studies on obstetric fistula in Tanzania have addressed issues related to social vulnerability [15, 22], experiences of birth care [12], perceived causes of obstetric fistula [23, 24], experiences of living with fistula [17, 25], religious coping of obstetric fistula [26] and women’s reintegration after fistula repair [27, 28]. This study uses women’s stories to illustrate their experience of failing to access adequate obstetric care and the challenges they faced during labour and delivery that caused an obstetric fistula. Understanding these experiences will guide the development of strategies to increase health facility deliveries in Tanzania.

### Model

The fundamental construct in understanding the cause of maternal deaths is the “three delays” model developed by Thaddeus and Maine [29]. These are (1) delay in recognising danger signs/decision to seek care, (2) delay in reaching a medical facility and (3) delay in receiving appropriate care once a facility is reached. We adopted the “three delays” model as a framework for describing women’s stories of their experiences of labour and delivery, particularly regarding how failure to promptly access adequate obstetric care led to the development of a fistula. This model provides a valuable theoretical foundation for examining composite interactions between women’s ability to make decisions on where to go for delivery, problems they encounter in reaching an adequate EmOC facility and receiving adequate obstetric care at the facility. In addition, it identifies key time periods in pregnancy and childbirth during which delays that have direct consequences on both maternal and neonatal survival can occur.

## Methods

This study uses narrative research, a qualitative approach on the propensity of humans to narrate experiences, and draws on data from a previous study on access to and quality of birth care in Tanzania: The problem of obstetric fistula and its implication conducted between 2010 and 2012 [30]. Stories provide a way for individuals to reflect on their experiences, create meaning, and imagine life’s possibilities [31]. They are concrete, personal and temporal accounts in which the narrator sets the scene and describes the main event and their resolution, including the central point of the story. In this study, stories from an in-depth analysis of women’s experience of labour and delivery provide a way to understand the complexities inherent in how women who live in rural settings of Tanzania access adequate obstetric care. By identifying the main story lines, a rich description of labour and delivery in the context of women’s lived experience of delays in accessing adequate obstetric care is provided [32]. The usefulness of stories in enriching our understanding of health and illness experiences has been recognised [31].

### Setting

As described elsewhere [25], the study was conducted at the Comprehensive Community Based Rehabilitation in Tanzania (CCBRT) hospital. CCBRT is a private, non-governmental organisation (NGO) in Dar es Salaam that serves as a major service delivery point for obstetric fistula repair in the Coastal region. It also receives patients from the central and eastern part of the country. The hospital has a 21-bed fistula ward, and a hostel where fistula patients live while awaiting fistula repair. It performs approximately 400 vesico-vaginal fistula (VVF) and recto-vaginal fistula (RVF) surgeries each year. The hospital has an active case finding program that traces patients in rural areas and brings them to the hospital for surgical treatment free of charge. This is done using a mobile phone based money transfer service to send cash to obstetric fistula patients for transportation costs to come to CCBRT. The program is facilitated by health care providers working in the primary health care facilities, who identify, communicate and facilitate transportation of women with obstetric fistula to CCBRT for treatment. The hospital also implements an incentive scheme for the identification of obstetric fistula cases.

### Participants

A convenience sample of 16 women affected by obstetric fistula was recruited for semi-structured interviews. It was essential to use convenience sampling because women were recruited from the fistula ward, and some were recruited after fistula repair. Therefore, their recruitment depended on their health condition. The inclusion criteria were women with fistula admitted to CCBRT for surgical repair (before or after fistula repair), ability to speak Kiswahili and willing to participate in the study. A senior nurse-midwife in the fistula ward aided in identifying women who met the inclusion criteria, explained the purpose and the method of the study, including principles of confidentiality, and arranged for a suitable time for an interview. All participants provided written informed consent.

### Data collection

Each woman participated in an audio recorded, semi-structured interview [33] that lasted about 1 h. During the interviews, women were encouraged to tell their stories of labour and delivery before they developed fistula. These interviews were conducted by the first author in the room adjacent to fistula ward. The interview room was quiet and out of sight and hearing range of other fistula patients and staff; this ensured the women’s privacy. The principle of saturation guided the sampling process. Saturation was achieved after 16 interviews, at which point answers from the women seemed to repeat information gained earlier and little new information was attained [34].

The semi-structured interview guide included topics and probing questions focussing on women’s experiences of labour and delivery (see Table 1). The interviewer used additional probing questions to clarify aspects of stories where necessary. Prompts and probes were used to encourage women to extend their responses and to provide stories that are more complete. Most of the participants spoke openly during all interviews.

**Table 1:** Semi-structured interview guide for women affected by fistula

1. Social and demographic background
— Before/After repair..........
— How old are you…………………
— What is your education level? Did you proceed after primary school?
— What do you do for a living?
— Where do you live?
— Who shares a house with you? Are you married?
2. Obstetric history
— How old were you when you got pregnant the first time? How many times have you been pregnant? How old were you when you got pregnant the last time? Was this last pregnancy your first pregnancy?
3. Can you please tell me about your last pregnancy?
— Probe: Did you attend antenatal clinic? Did you get the services you expected? Where did you plan to give birth? How far was it from your home? How did you plan to get there? Did you have any problems during your pregnancy? Who did you seek advice from during pregnancy?
4. Can you please tell me about the last time you gave birth?
— Probe: How long did it take from the onset of labour pains until the child was out? Where were you when labour started? How long did it take you to seek health care after you started labour? What were the reasons for delay? Where did you go first? Who made a decision as to where to go to seek help? What were the constraints/barriers/problems in the process of seeking birth care? Where did you finally deliver? How was your delivery conducted? Who assisted you? Who was present during labour? When you or the ones that assisted you realised that the baby did not come out as expected? What action was taken? What happened after the delivery? Did you go home? Did you remain in the hospital?

### Data analysis

Data analysis was guided by recommendations for the identification and interpretation of narratives [35]. The audio recorded interviews were transcribed and read several times to identify the parts of the interview that made up each woman’s story. In this stage, the audio recorded interviews were reviewed to verify the written/transcribed interviews. Relevant sections of the interview were marked accordingly. These were all texts that described women experiences of delay in accessing obstetric care. The interviews were re-read to capture the universal impression of each woman’s story. To facilitate this, the author prepared brief summaries of each woman’s narrative, highlighting general impressions, as well as unusual features, of each story. The authors systematically reviewed summaries of the stories to identify the central narrative or story lines that women used to explain their experiences of being unable to access obstetric care during labour and delivery (See Table 2). Decisions were made through consensus and in cases of disagreements, the authors returned to the transcript to ensure interpretations were grounded in the data. Using these central narratives, the interviews were re-read and coded.

**Table 2:** Example of the process of analysis

Summaries of women’s narratives	Central narrative or story line
“I was advised by nurses to go to the major hospital. However, the day labour pains started my father had no cash.”	Lacking money to get to health facilities
“We did not have cash, fare”	
“I was asked to deliver at the hospital but it was not possible because I did not have cash and I did not know where I would get the money to go there.”	
“We did not have money.”	
“My husband did not secure enough money.”	

The authors then probed each central narrative [35], whereby the process began by reading all relevant coded segments. Attention was given to the identification of important components of the stories, what was emphasized and the similarities and differences in the way the stories were told. All texts from the interviews were first analysed for identification of delay patterns in accessing obstetric care. Women’s narratives were organised according to the “three delays” model [29]. The research team crosschecked analysis and discussed and agreed on the sorting of codes and naming of categories.

## Results

The 16 women who participated in this study were between 19 and 43 years of age. Most of the women lived in rural areas (82 %) and had no or primary education (88 %). All of the women were unemployed (See Table 3).

**Table 3:** Characteristics of women affected by obstetric fistula

Woman	Age	Marital status	Years in school	Duration with fistula	Domicile
1	19	Single	7	4 months	Matombo, Morogoro
2	25	Married	3	>1 year	Handeni, Tanga
3	35	Single	7	18 years	Kisongo, Kilwa
4	20	Divorced	7	6 months	Mlandizi, Pwani
5	28	Married	12	2 months	Temeke, Dar es Salaam
6	30	Married	12	3 months	Iringa road, Iringa
7	33	Divorced	7	19 years	Mbori, Dodoma
8	29	Divorced	0	10 years	Ifakara, Morogoro
9	35	Married	7	2 years	Manyoni, Singida
10	40	Married	7	1 year	Mpwapwa, Dodoma
11	43	Separated	7	20 years	Rundi, Dodoma
12	40	Single	7	20 years	Manyoni, Singida
13	25	Divorced	7	8 months	Iringa
14	28	Divorced	7	12 years	Mvumi, Dodoma
15	29	Divorced	0	9 years	Kibakwe, Dodoma
16	18	Single	0	3 months	Mseta, Dodoma

In the analysis of women’s experiences of labour and delivery and reasons for their inability to access adequate obstetric care in a timely manner, four (4) general story lines were identified: (1) failing to decide on a health care facility for delivery, (2) lacking money to get to a health care facility, (3) lacking transportation to a health care facility and (4) lacking quality birth care at the health care facility.

### Failing to decide on a health care facility for delivery

The basic story line in the narrative of failing to decide for health facility delivery is:“usually in the village decisions on where to go for delivery is made by husbands, and if you are not married or you are without a partner, elders and parents, make decision on when and where to go for delivery”. In this study, husbands, mothers-in-law, uncles and grandmothers made decisions on where women could seek obstetric care.

Women who delivered at home had attended antenatal clinics regularly for check-ups and intended to give birth in the health care facility. However, because their husbands and mothers-in-law had decided that the women should give birth at their homes, they had no other option. Husbands and mothers-in-law made decisions for home deliveries due to costs and customs. Most of women started labour while their husbands were not around, and therefore, their mothers-in-law and uncles made the decisions to wait and give birth at home with the assistance of traditional birth attendants (TBA), who are usually present in the village and were well known.

“… My mother told my uncle that I am sick, and then he said that she (mother) has to call the traditional birth attendant” (Single, aged 18, Mseta-Dodoma).

“… My mother-in-law said I should wait as I could deliver at home. You know in the village people do deliver in homes” (Single, aged 35, Kisongo-Kilwa).

### Lacking money to get to a health care facility

The basic story line of lacking money to get to a health care facility within the women’s narratives occurred as follows: “I was advised by nurses to go to the major hospital. However, the day labour pains started my father had no cash…”. “We did not have cash, fare…”. Many women who participated in this study resided in remote rural areas. In these areas, health care facilities providing EmOC are far away and public transportation is limited. When transportation is available, women have to spend money to reach to an adequate health care facility for delivery, a cost that is too expensive for an ordinary woman in the village to afford. Therefore, women were unable to go to the health facilities because they did not have enough money to pay for transportation costs. Some women admitted that despite being informed during ANC visits to go to a health care facility for delivery, they gave birth at home because they lacked money for transportation:

“I was asked to deliver at the hospital but it was not possible because I did not have cash and I did not know where I would get the money to go there. As peasants, our income as you know are very little … we did not have money” (Married, age 35, Manyoni-Singida).

“… it is only that my husband did not secure enough money” (Married, aged 40, Mpwapwa, Dodoma).

The bus fare was also too expensive for an ordinary woman who lived in a rural area to afford:

“… It is very far. You cannot walk. You have to take a bus, and the bus fare is 5500 shillings and if you are two it will be 11,000/= (about $ 5)” (Married, aged 35, Manyoni-Singida).

“From the village where we lived is about 178 kilometres and the bus fare is expensive, about 21,000 Tshs (about $10) we could not afford” (Married, aged 25, Handeni-Tanga).

### Lacking transportation to a health care facility

Lack of money was not the only reason for women giving birth at their homes. Women were unable to go to the health facilities for birth care because they could not get transportation to these health care facilities:

“… labour pains started while I was at home which is far, … there were no means of transport, even bicycles or push carts. … it was not possible for me to walk to the hospital when the baby’s head was already protruding out …” (Married, aged 35, Manyoni-Singida).

Others got to the health facility after substantial delay:

“We had to cross the river by boat, it took us 8 h to get to main hospital by then the baby was already dead… (crying…)” (Single, aged 19, Matombo-Morogoro).

Because of lack of transportation or the inability to pay for transportation costs, some women had to walk for many hours to get to the nearest health care facility providing CEmOC:

“It took long time to get to the hospital … I am not sure, may be 8 h” (Separated, aged 43, Rundi-Dodoma).

“The hospitals are far. We had to walk…It was a long walk, after a 2-hour hard walk along the way, I felt as if something had ruptured in the abdomen and immediately started vaginal bleeding” (Divorced, age 28, Mvumi-Dodoma).

### Lacking quality birth care at the health care facility

The availability of skilled attendant personnel is pivotal for the provision of high quality health care. Women in this study reported that doctors in the primary health care facilities were not available to refer women to the health care facilities providing EmOC when labour did not progress well. A woman who lived with obstetric fistula for 19 years told her story of having to wait in the health care facility for 24 h before the doctor could see her:

“When we got to the dispensary nurses told me to wait. At 8 pm labour pains became intense, I started pushing but the baby could not come out, and the doctor was not around. Next day I continued pushing the whole day again until at around 8 pm when the doctor came, he inserted his hand and started pulling the baby, sadly only the head came out, the entire body was left in the womb, thats when he asked us to go to the big hospital. In this hospital there was no transport (ambulance) therefore my relatives hired a tractor at a cost of 30,000/- shillings (about 15$) that took me to the big hospital. I travelled with the baby’s head out for 4 h all the way to the hospital. In the big hospital doctors pulled the baby using instruments. After pulling hard for long, the baby was removed, then my pelvis relaxed completely. Soon after, both urine and feaces started leaking through the vagina”. … If I got early to this hospital it would have been different, … nurses in the village health facility were certain that I will deliver, they kept on saying wait, wait while the possibility of giving birth normally was minimal (crying…)” (Divorced, aged 33, Mbori-Dodoma).

Other women reported experiences of waiting for a long time in the primary health care facility because nurses did not make decisions to consult with them or timely transfer them to an CEmOC health care facility in a timely manner after recognizing that spontaneous delivery was difficult:

“…In the health facility I spend the night until morning … I had pains, the day passed, I slept again until morning again, and it was when a decision was made to transfer me to another hospital. They said it was because I had urine retention. On the third day is when I was transported to a big hospital” (Divorced, aged 20, Mlandizi-Pwani).

“…I stayed for a long time, and each time I called for help, the nurse would tell me to lie on my side. I continued to lie on one side until midday. Since 10 am they told me I will deliver at 2 pm. Later, my mother asked them to let us go to the main hospital, they refused saying I will deliver because membranes had ruptured already, … but I could not” (Divorced, aged29, Ifakara-Morogoro).

Another woman who was a primigravida spent 4 days labouring in the primary health care facility before the referral decision was made:

“… it took 4 days at the village health facility, I could not give birth and then I was referred to the big hospital” (Divorced, aged 29, Kibakwe, Dodoma).

## Discussion

Access to timely and quality care during labour and delivery is of paramount importance in reducing women’s and neonates’ mortality rates [29]. Women in this study were unable to access quality birth care because of their inability to make decisions for health care facility delivery. Health care facilities providing CEmOC were located far from where the women lived. Therefore, women were unable to meet transportation costs, and in some villages, public transportation was unavailable. However, women who did reach the health care facilities reported receiving low quality birth care.

Determining where a woman is to give birth is important and requires advanced planning during the pregnancy [36]. In this study, decisions on where women should go for birth care were made when the women were already in labour. It is ideal to decide on where a woman should give birth earlier in the pregnancy. To facilitate swift care-seeking behaviours for maternal health services, this decision should be known by the rest of the family. Husbands and mothers-in-law had a significant role in deciding on a place where women should give birth. This is not surprising, because in Tanzania, as is the case in other African countries, the dominant social structure is patriarchal, with the paternal side holding power in most decision-making processes [37]. This applies even to the birthing process, where the paternal family has the final say. Other studies have also documented the powerlessness of women in societies where they are usually not involved in the decisions to seek birth care either from a TBA or at a health care facility [38]. Women’s failure to access adequate obstetric care at the health care facilities could be explained by their inability to make decision by themselves, living far away from health care facilities that provide CEmOC and their inability to meet transportation costs. These findings complement those of previous studies [39, 40].

Women’s lack of decision-making power is commonly associated with their low socio-economic status, young age, illiteracy and unemployment [12, 39–43]. Women who participated in this study were either illiterate or had not gone beyond completing primary education. In addition, they were involved in unskilled, low-paying jobs or in subsistence farming. Therefore, these women had no cash of their own for emergencies unless their husbands or relatives gave it to them. Thus, money is an important determinant for accessing quality obstetric care [44].

In Tanzania, more than 90 % of pregnant women attend antenatal clinics for check-ups. It is expected that birth preparedness, a major components in focused antenatal care, is taught during these visits. However, there are several reasons why women do not prepare for birth [45]. One reason might be that women do not acquire adequate knowledge on birth preparedness during antenatal care visits, or the knowledge given was of sub-standard. In addition, knowledge does not always influence practice [46]. Birth preparedness ensures positive outcomes for the mother and her newborn. Key elements of birth preparedness include attending the antenatal clinic for check-ups at least 4 times during pregnancy, identifying and making a plan for reaching an adequate health care facility during labour (i.e., setting aside personal funds to cover the costs of travelling to the health facility, delivering with a skilled birth attendant). Furthermore, recognising signs of complications (i.e., knowing what community resources, such as emergency transport, funds and communications, are available in the case of emergencies) and having a plan for emergencies [36].

Consistent with other studies, women in our study failed to get to a health care facility for adequate birth care in a timely manner because of the distance to the health facility, poor road conditions, inadequate or inappropriate transport, inability to pay for transportation or because public transportation was unavailable [12, 42, 47–51]. With the government’s goal of increasing access to health care services by making sure that each village has a health care facility [52, 53], these primary health care facilities have no components of EmOC to manage obstetric emergencies [54, 55]. Therefore, women must walk long distances to the health care facilities that provide CEmOC.

Approximately 80 % of maternal deaths could be prevented if, at the time of delivery, women have timely access to relatively basic maternal services and are attended to by skilled personnel [56]. A skilled attendant (a midwife or a doctor) would continuously monitor women’s progress of labour using a partograph to facilitate timely decision-making and referral, which are key factors in preventing obstructed labour and thereby preventing obstetric fistula [57]. Carelessness and lack of capacity to identify and manage labour related complications, lack of communication mechanisms and lack of ambulance services that can facilitate efficient referral of women to the CEmOC health care facilities once the decision is made contribute to women’s inability to access adequate obstetric care in a timely manner [58]. This calls for the government to increase the production of skilled attendants and upgrading primary health care facilities to provide comprehensive emergency obstetric care to ensure that all women, regardless of where they reside, can access emergency obstetric services. This will also ensure equity in health care services provision, which is a human rights agenda [59].

Getting to the health care facilities does not necessarily mean the end of the health care seeking journey. If the facility is not well equipped to handle obstetric emergencies, or does not have adequately skilled personnel to make diagnoses and swift decisions, women would still receive inadequate obstetric care. The health care system in Tanzania is organised in a referral pyramid, starting with dispensaries at the bottom and rural health centres (RHCs) that provide basic emergency obstetric care (BEmOC) and treatment of minor conditions. At the district level, there are district hospitals at the first referral level, where necessary drugs, equipment and skilled staff are available to provide CEmOC. There are also regional hospitals in each region, with the highest levels being national and specialised hospitals [53]. Although all health care facilities in Tanzania, by design, need to provide EmOC based on the level of the facility, only those of District, Regional and National hospitals provides CEmOC. Further, health care facilities in rural Tanzania suffer from critical shortages of skilled personnel that negatively affect the quality of care provided [11, 60]. It is common to find only one nurse-midwife working in a rural health care facility serving about 10,000 people [54, 61]. When this health care provider falls sick, there will be no one left to provide care.

Because of the shortage of skilled personnel, especially in the primary health care facilities, these personnel lack professional support and have no one with whom to consult when things become difficult; thus, they are forced to get assistance from persons without any training in midwifery skills [61]. Skilled attendance is one of the key factors in reducing maternal mortality and morbidity [1]. Skilled attendants are people with midwifery skills who have been trained to proficiency in the skills necessary to manage normal pregnancies, childbirth, and the immediate postnatal period, and to identify, manage or refer women and newborns with complications [42].

Women’s inability to receive adequate birth care indicates a failure of the health care delivery system, which prohibits continuity of maternity care and is a major contributing factor for maternal mortality [56, 62]. Studies of Tanzania’s health care system have reported inadequacies of the health care facilities, which experience shortages of motivated, competent staff with the right attitude [61, 63]; shortages of equipment, materials, and supplies [64]; and poor referral systems [24]. Women in this study received inadequate birth care while they are already at the health care facility. For example, some women were asked to wait for a long time before a referral decision was made, simply because nurses failed to diagnose obstructed labour and consult, or the doctor was not around to refer a woman for comprehensive obstetric care. When women have a choice, they will go to health care facilities they perceive to provide better quality of care, regardless of distance [65]. Access to a well-equipped health care facility may be more important than other factors in determining whether a woman seeks birth care. However, such access does not completely eradicate inefficiencies in the provision of birth care, unless skilled attendants are well motivated to perform their duties.

## Conclusion

Women’s lack of decision-making power, long distances to health care facilities providing CEmOC, lack of money and unavailability of transportation were the primary barriers for women to give birth in health care facilities. Nevertheless, waiting for a long time in the health care facilities before the decision to refer or consult was another problem, which caused women not to access quality birth care in a timely manner. Thus, empowering women socially and financially could promote health care facility deliveries. Tanzania’s government must upgrade primary health care facilities to provide CEmOC and increase incentives and the number of skilled personnel for women to access quality birth care. This may result in women’s improved birth care experiences and reducing maternal and neonatal morbidity and mortality.
